# Supplementary material for: Preclinical development of a stabilized RH5 virus-like particle vaccine that induces improved antimalarial antibodies
Source: Cell Rep Med. 2024 Jul 16;5(7):101654. doi: 10.1016/j.xcrm.2024.101654 (PMC11293324; doi:10.1016/j.xcrm.2024.101654)
Supplement: Document S1. Figures S1–S4 [file mmc1.pdf]

Cell Reports Medicine, Volume *5*

Supplemental information

Preclinical development of a stabilized RH5 virus-like particle vaccine that induces improved antimalarial antibodies

Lloyd D.W. King, David Pulido, Jordan R. Barrett, Hannah Davies, Doris Quinkert, Amelia M. Lias, Sarah E. Silk, David J. Pattinson, Ababacar Diouf, Barnabas G. Williams, Kirsty McHugh, Ana Rodrigues, Cassandra A. Rigby, Veronica Strazza, Jonathan Suurbaar, Chloe Rees-Spear, Rebecca A. Dabbs, Andrew S. Ishizuka, Yu Zhou, Gaurav Gupta, Jing Jin, Yuanyuan Li, Cecilia Carnrot, Angela M. Minassian, Ivan Campeotto, Sarel J. Fleishman, Amy R. Noe, Randall S. MacGill, C. Richter King, Ashley J. Birkett, Lorraine A. Soisson, Carole A. Long, Kazutoyo Miura, Rebecca Ashfield, Katherine Skinner, Mark R. Howarth, Sumi Biswas, and Simon J. Draper

Supplementary Figures

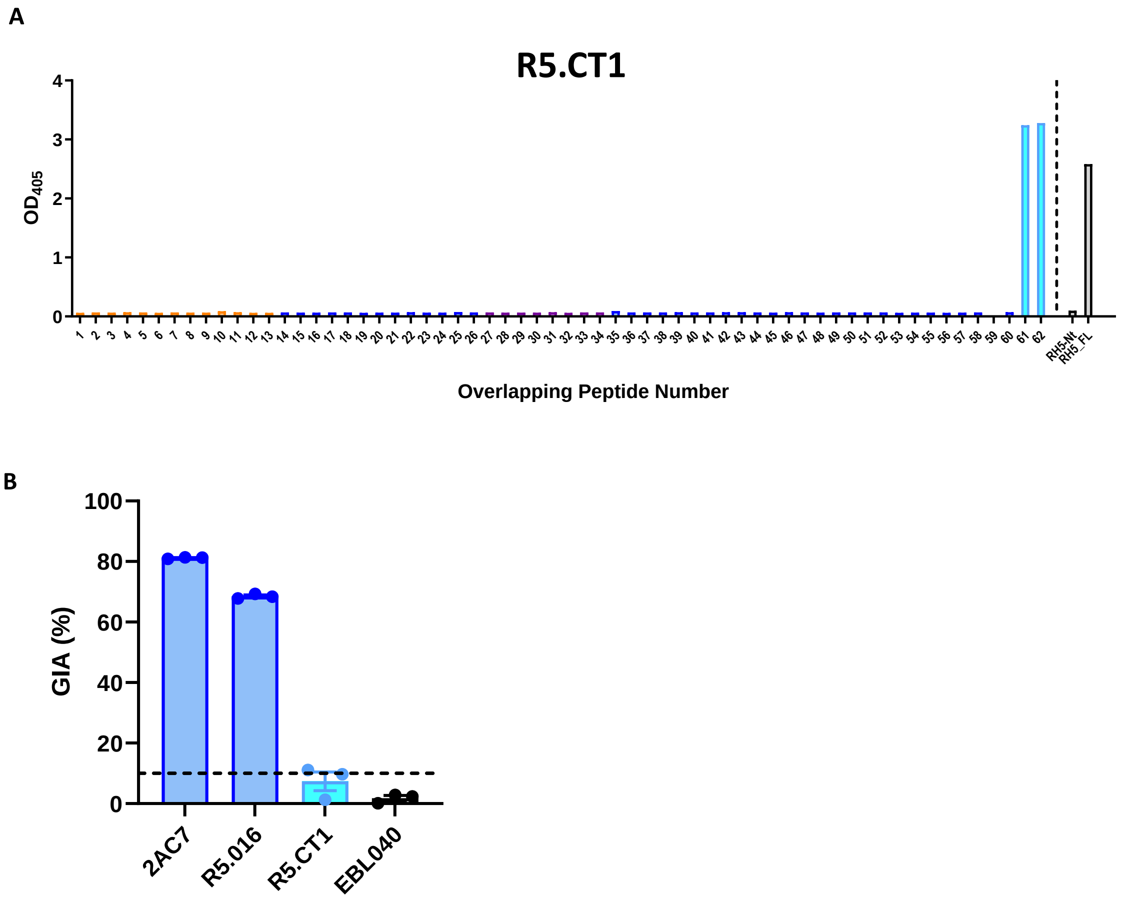

Figure S1. Assessment of an anti-RH5 C-terminal human mAb; related to Figure 1.

(**A**) The recombinant human IgG1 mAb, R5.CT1, was tested by ELISA at 2 µg/mL against linear overlapping peptides spanning the RH5 vaccine insert, colour-coded as per **Figure 1**. Data from single wells are shown, but data are representative of N=3 repeats. Peptides 61 and 62 span the C-terminal 20 amino acids of RH5 and differ by only one amino acid [1]. RH5-Nt and RH5_FL = recombinant protein controls for RH5 N-terminus and full-length, respectively. (**B**) Individual mAbs were tested in triplicate in the GIA assay against 3D7 clone *P. falciparum* parasites. Individual and mean ± SEM GIA % are shown for each mAb. 2AC7 and R5.016 (positive control mAbs) bind RH5ΔNL [2,3] and were tested at 15-20 µg/mL; EBL040 (negative control mAb against Ebola virus) [4] and R5.CT1 were tested at 0.5 mg/mL. Dashed line at 10 % GIA represents typical cut-off for positivity in the assay.

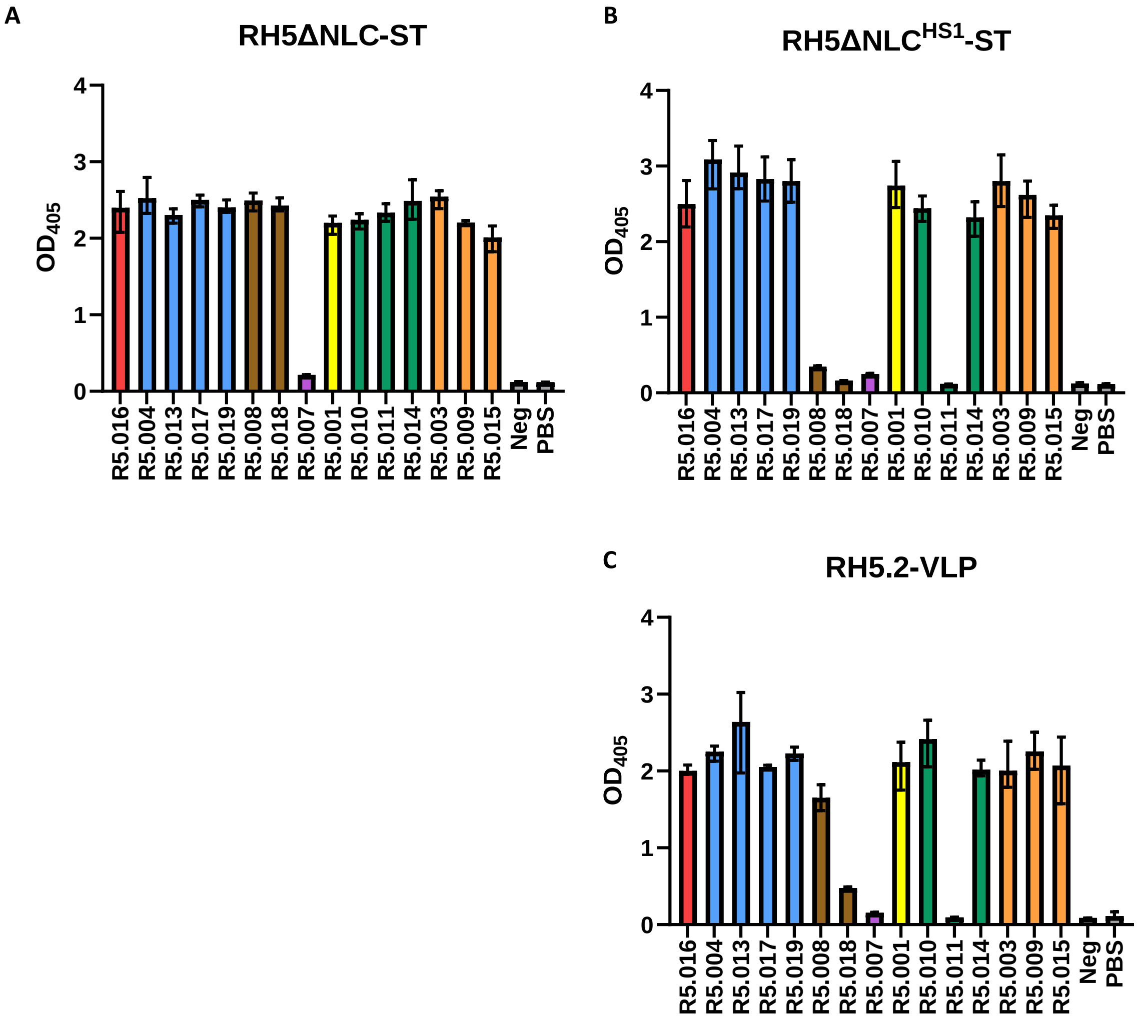

Figure S2. ELISA to screen for RH5 protein binding to a panel of anti-RH5 human mAbs; related to Figures 2 and 3.

A binding ELISA was performed on (**A**) RH5ΔNLC-ST protein, (**B**) RH5ΔNLC^{HS1}-ST protein and (**C**) RH5.2-VLP using a panel of anti-RH5 human mAbs. This mAb panel is color-coded as previously reported and defines seven epitope regions or antibody competition binding groups across the RH5 molecule [2]. Antibodies of the same color compete for binding, but do not compete with antibodies in other color-coded groups. Clone R5.007 (purple) binds a linear peptide epitope in the intrinsic loop [2] and therefore should not bind to either of these proteins given they lack this sequence. The remaining six groups bind conformational epitopes [2]. The red, blue and brown groups include growth inhibitory antibodies that bind close to or within the basigin binding site on RH5 [2]; the green antibodies do not inhibit invasion but can synergize with other growth inhibitory antibodies [2]; the

yellow and orange antibodies do not inhibit parasite growth *in vitro* but block RH5 binding to CyRPA [2,5]. Neg is an irrelevant human IgG1 antibody control. PBS = phosphate-buffered saline only control. Results show the mean and range of optical density at 405 nm (OD_{405}) of triplicate wells.

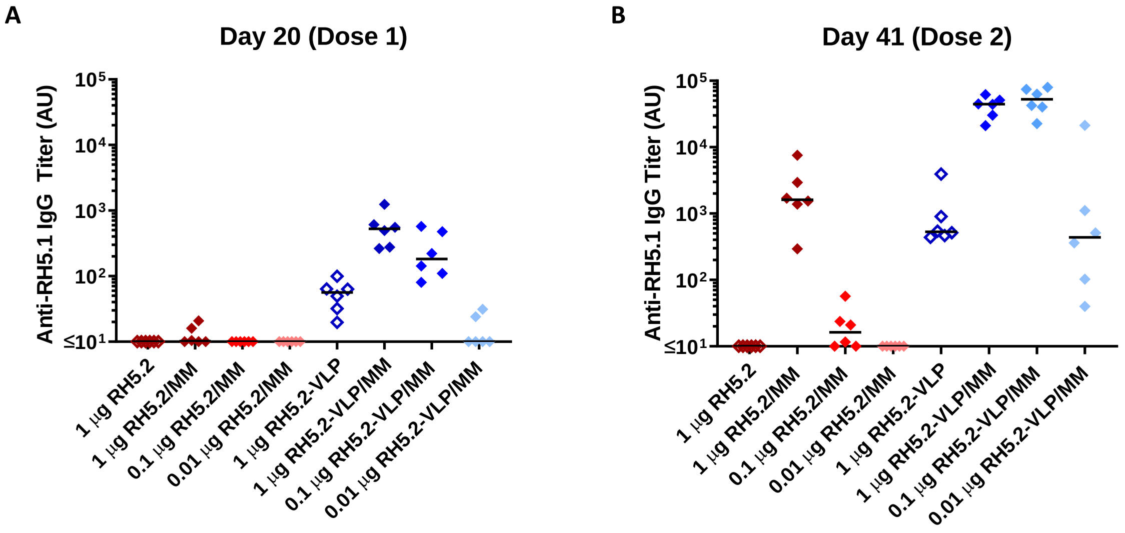

Figure S3. Immunogenicity testing of the RH5.2-VLP vaccine candidate; related to Figure 3.

BALB/c mice (N=6 per group) were immunized intramuscularly with three doses of RH5.2-ST protein or RH5.2-VLP on days 0, 21 and 42 either with (closed symbols) or without (open symbols) Matrix-M™ (MM) adjuvant. Dosing of the RH5.2-VLP was adjusted in each case to deliver the same molar amount of RH5.2 antigen as the soluble protein comparator (1, 0.1 or 0.01 µg). Anti-RH5 (full-length RH5.1) IgG titers were measured in the serum by ELISA after (**A**) dose 1 at day 20, and (**B**) dose 2 at day 41. Each point represents a single mouse and the line the median.

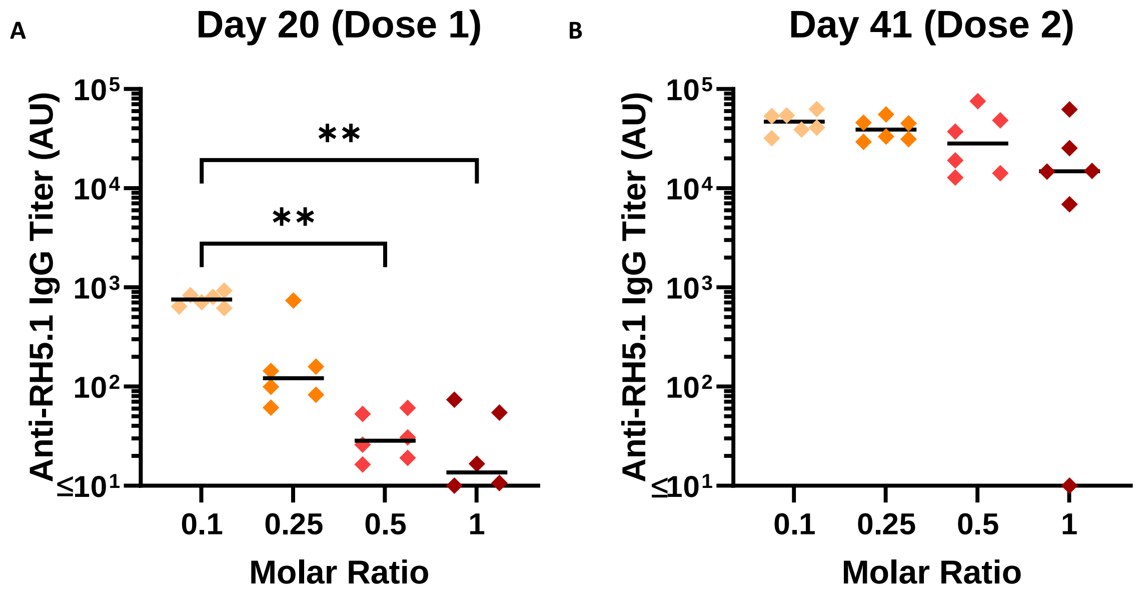

Figure S4. Immunogenicity testing of the RH5.2-VLP vaccine produced with different conjugation efficiencies; related to Figure 3.

BALB/c mice (N=6 per group) were immunized intramuscularly with three doses of RH5.2-VLP, produced using the indicated molar ratios of RH5.2-ST to HBsAg-SC (0.1:1, 0.25:1, 0.5:1 and 1:1), on days 0, 21 and 42. Dosing was adjusted in each case to deliver the same molar amount of RH5.2 antigen (10 ng); total RH5.2-VLP dose = 232, 52, 40 and 23 ng, respectively. All vaccines were formulated in Matrix-M™ adjuvant. Anti-RH5 (full-length RH5.1) IgG titers were measured in the serum by ELISA after (**A**) dose 1 at day 20, and (**B**) dose 2 at day 41. Each point represents a single mouse and the line the median. Analysis using Kruskal-Wallis test with Dunn's multiple comparison test across the four groups; ***P* < 0.01.

Supplementary References

1. Payne, R.O., Silk, S.E., Elias, S.C., Miura, K., Diouf, A., Galaway, F., de Graaf, H., Brendish, N.J., Poulton, I.D., Griffiths, O.J., *et al.* (2017). Human vaccination against RH5 induces neutralizing antimalarial antibodies that inhibit RH5 invasion complex interactions. *JCI Insight* 2, 96381.
2. Alanine, D.G.W., Quinkert, D., Kumarasingha, R., Mehmood, S., Donnellan, F.R., Minkah, N.K., Dadonaite, B., Diouf, A., Galaway, F., Silk, S.E., *et al.* (2019). Human Antibodies that Slow Erythrocyte Invasion Potentiate Malaria-Neutralizing Antibodies. *Cell* 178, 216-228.
3. Douglas, A.D., Williams, A.R., Knuepfer, E., Illingworth, J.J., Furze, J.M., Crosnier, C., Choudhary, P., Bustamante, L.Y., Zakutansky, S.E., Awuah, D.K., *et al.* (2014). Neutralization of Plasmodium falciparum Merozoites by Antibodies against PfRH5. *J Immunol* 192, 245-258.
4. Rijal, P., Elias, S.C., Machado, S.R., Xiao, J., Schimanski, L., O'Dowd, V., Baker, T., Barry, E., Mendelsohn, S.C., Cherry, C.J., *et al.* (2019). Therapeutic Monoclonal Antibodies for Ebola Virus Infection Derived from Vaccinated Humans. *Cell Rep* 27, 172-186 e177.
5. Ragotte, R.J., Pulido, D., Lias, A.M., Quinkert, D., Alanine, D.G.W., Jamwal, A., Davies, H., Nacer, A., Lowe, E.D., Grime, G.W., *et al.* (2022). Heterotypic interactions drive antibody synergy against a malaria vaccine candidate. *Nat Commun* 13, 933.
